# Disturbance and nutrients synchronise kelp forests across scales through interacting Moran effects

**DOI:** 10.1111/ele.14066

**Published:** 2022-06-30

**Authors:** Max C. N. Castorani, Tom W. Bell, Jonathan A. Walter, Daniel C. Reuman, Kyle C. Cavanaugh, Lawrence W. Sheppard

**Affiliations:** ^1^ Department of Environmental Sciences University of Virginia Charlottesville Virginia USA; ^2^ Department of Applied Ocean Physics & Engineering Woods Hole Oceanographic Institution Woods Hole Massachusetts USA; ^3^ Earth Research Institute University of California Santa Barbara California USA; ^4^ Department of Ecology and Evolutionary Biology University of Kansas Lawrence Kansas USA; ^5^ Center for Ecological Research University of Kansas Lawrence Kansas USA; ^6^ Laboratory of Populations Rockefeller University New York New York USA; ^7^ Department of Geography University of California Los Angeles California USA; ^8^ Marine Biological Association of the United Kingdom Plymouth UK

**Keywords:** coherence, disturbance, Moran effect, nitrate, North Pacific Gyre Oscillation, oceanography, population dynamics, remote sensing, spatial synchrony, wavelet transforms

## Abstract

Spatial synchrony is a ubiquitous and important feature of population dynamics, but many aspects of this phenomenon are not well understood. In particular, it is largely unknown how multiple environmental drivers interact to determine synchrony via Moran effects, and how these impacts vary across spatial and temporal scales. Using new wavelet statistical techniques, we characterised synchrony in populations of giant kelp *Macrocystis pyrifera*, a widely distributed marine foundation species, and related synchrony to variation in oceanographic conditions across 33 years (1987–2019) and >900 km of coastline in California, USA. We discovered that disturbance (storm‐driven waves) and resources (seawater nutrients)—underpinned by climatic variability—act individually and interactively to produce synchrony in giant kelp across geography and timescales. Our findings demonstrate that understanding and predicting synchrony, and thus the regional stability of populations, relies on resolving the synergistic and antagonistic Moran effects of multiple environmental drivers acting on different timescales.

## Graphical abstract

Spatial synchrony is a ubiquitous feature of population dynamics, but it is largely unknown how multiple environmental drivers interact to determine synchrony via Moran effects, and how these impacts vary across spatial and temporal scales. Using new wavelet statistical techniques, we characterized synchrony in populations of giant kelp, a widely distributed marine foundation species, and related synchrony to variation in oceanographic conditions. We discovered that disturbance and resources—underpinned by climatic variability—act individually and interactively to produce synchrony across geography and timescales, demonstrating that predicting regional population stability relies on resolving the synergistic and antagonistic Moran effects of multiple environmental drivers acting on different timescales.

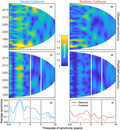

## INTRODUCTION

A fundamental feature of population dynamics is spatial synchrony, the tendency for populations in different locations to exhibit correlated fluctuations over time (Moran, 1953). Spatial synchrony (hereafter, ‘synchrony’) is ubiquitous, having been observed in a wide range of taxa and over scales of centimetres to thousands of kilometres (Liebhold et al., 2004). Synchrony is important to population dynamics because it influences regional population persistence, stability, and resilience. Local population fluctuations (those of populations in different locations) that are *asynchronous* compensate for each other, whereas those that are *synchronous* reinforce each other, increasing population variance at the regional scale. Hence, strong synchrony increases temporal variability of regional abundance, which can reduce stability and increase extinction risk (Descamps et al., 2013; Hanski & Woiwod, 1993; Heino et al., 1997; Ojanen et al., 2013). Moreover, these effects can cascade to community dynamics and biodiversity (Cattadori et al., 2005; Haynes et al., 2009; Kent et al., 2007; Satake et al., 2004; Walter et al., 2020; Walter, Shoemaker, et al., 2021). Due to its pervasiveness and significance, understanding the patterns, causes, and consequences of synchrony is a key goal in ecology and its applications in conservation (Earn et al., 2000; Tack et al., 2015), agriculture (Sheppard et al., 2016; Walter et al., 2020), forestry (Haynes et al., 2018; Peltonen et al., 2002), wildlife management (Post & Forchhammer, 2002, 2004), and epidemiology (Earn et al., 1998).

Despite the importance of synchrony, three major aspects remain poorly understood: (1) Populations may be synchronised to different extents on different timescales (i.e. periods of fluctuations, such as annual or decadal) or during specific, transient periods (Keitt, 2008; Vasseur et al., 2014; Walter et al., 2020; Walter, Hallett, et al., 2021), but traditional approaches often ignore or misidentify such temporal complexity (Anderson et al., 2019, 2021; Defriez et al., 2016; Desharnais et al., 2018; Sheppard et al., 2016, 2019). (2) Synchrony can differ regionally, but most investigations overlook geographical patterns in synchrony and their underlying drivers (Anderson et al., 2019; Koenig et al., 2017; Walter et al., 2017, 2022). (3) The relative influence of multiple drivers of synchrony and—most importantly for this study—their interactions are still understudied (Ranta et al., 1995; Sheppard et al., 2019; Walter et al., 2017) outside of laboratory experiments with microorganisms (Duncan et al., 2015; Fox et al., 2011; Thompson et al., 2015; Vogwill et al., 2009) and a few well‐described real populations (e.g. defoliating moths; Walter et al., 2017; Haynes et al., 2019). Spatially correlated environmental fluctuations can synchronise populations across space in a phenomenon known as the ‘Moran effect’ (Moran, 1953). In theory, many environmental processes can induce Moran effects concurrently, but the separate and combined effects of multiple Moran drivers, as well as whether these differ across spatial and temporal scales, are little studied. Recently, Sheppard et al. (2019) demonstrated for marine phytoplankton that drivers of synchrony can interact synergistically, producing more synchrony than would be expected through independent additive effects. It remains unknown how widespread and important interactive Moran effects are, or whether antagonistic Moran interactions can dampen synchrony, but it is reasonable to suspect that interactions are common because most species are influenced by multiple environmental drivers, which are often spatially autocorrelated.

Resolving these gaps in understanding is urgent in light of accelerating global change. Changes in synchrony are associated with climatic variation (Allstadt et al., 2015; Cattadori et al., 2005; Hansen et al., 2013; Kahilainen et al., 2018; Ong et al., 2016; Post & Forchhammer, 2002, 2004) and recent studies suggest that some systems are becoming more or less synchronous in association with climate trends (Defriez et al., 2016; Di Cecco & Gouhier, 2018; Koenig & Liebhold, 2016; Ojanen et al., 2013). The degree to which climate shifts cause changes in synchrony remains underexplored but is now recognised as likely to be important (Hansen et al., 2020; Özkan et al., 2016). Changing interactions between Moran drivers have the potential to be a crucial but unrecognised means by which climate change alters population synchrony and stability.

Here, we examined spatial and temporal patterns of synchrony in a broadly distributed marine foundation species, giant kelp *Macrocystis pyrifera*, using a 33‐year spatial time series of canopy biomass spanning >900 km of coastline in California, USA. The outstanding availability of biological and oceanographic datasets in our study region enabled us to overcome the typical challenges to resolving the patterns and drivers of synchrony. We used wavelet techniques to quantify time‐ and timescale‐specific patterns of synchrony, and how these varied geographically. We then applied newly developed multivariate wavelet regression models to investigate how disturbance (storm‐driven waves) and resources (seawater nutrients) act individually and interactively to structure giant kelp synchrony, and whether the importance of these forces varies across timescales and geography. In doing so, we accomplish three goals: (1) quantify the timescale structure of giant kelp synchrony and identify the main causes of synchrony; (2) demonstrate that multiple environmental factors can combine to produce timescale‐specific synchrony via synergistic or antagonistic Moran effects, thereby providing evidence that the new mechanism of interactions between Moran effects is potentially widespread; and (3) show that the influence of individual and interactive synchrony drivers can differ strongly across geography and timescales.

## MATERIAL AND METHODS

### Study system

Giant kelp is the largest and most widely distributed kelp species, forming highly productive forests that define much of the community and ecosystem dynamics on shallow rocky reefs within its range (Castorani et al., 2018, 2021; Schiel & Foster, 2015). Giant kelp populations are well‐suited for studying synchrony because they are exceptionally dynamic relative to most foundation species. Short lifespans of plants (usually 2–3 years) and fronds (1–6 months) combine with short generation times (about one or more per year) and rapid growth (~2% per day) such that standing biomass turns over 6–12 times per year (Rassweiler et al., 2018; Reed et al., 2008, 2011; Rodriguez et al., 2013). Hence, giant kelp responds quickly to changing environmental conditions (Bell, Cavanaugh, Reed, & Siegel, 2015; Edwards & Estes, 2006; Parnell et al., 2010).

Nearshore oceanographic conditions that influence giant kelp dynamics may induce synchrony via Moran effects (Cavanaugh et al., 2013; Parnell et al., 2010). Storm‐driven waves may synchronise populations as a major source of mortality (Bell, Cavanaugh, Reed, & Siegel, 2015; Dayton et al., 1992; Reed et al., 2011) that can extirpate kelp locally when particularly severe (Edwards & Estes, 2006; Graham et al., 1997; Young et al., 2016). Seawater nutrients may also be important for synchrony because they fuel rapid growth (Kopczak et al., 1991; Zimmerman & Kremer, 1984, 1986), while prolonged periods of low nutrients are associated with declines (Bell, Cavanaugh, Reed, & Siegel, 2015; Dayton et al., 1999; Edwards, 2004; Ladah et al., 1999; Parnell et al., 2010), inhibited recruitment (Deysher & Dean, 1986; Hernández‐Carmona et al., 2001) and delayed recovery (Cavanaugh et al., 2011; Edwards & Hernández‐Carmona, 2005). Nutrients are supplied primarily through upwelling of cool, nitrate‐rich waters (Huyer, 1983; McPhee‐Shaw et al., 2007), which is partly driven by large‐scale, interannual to decadal climate cycles (Di Lorenzo et al., 2008, 2013). Climate change may bring about stormier seas with prolonged periods of nutrient depletion that could increase kelp losses (Butler et al., 2020; Cavanaugh et al., 2019).

**FIGURE 1:**
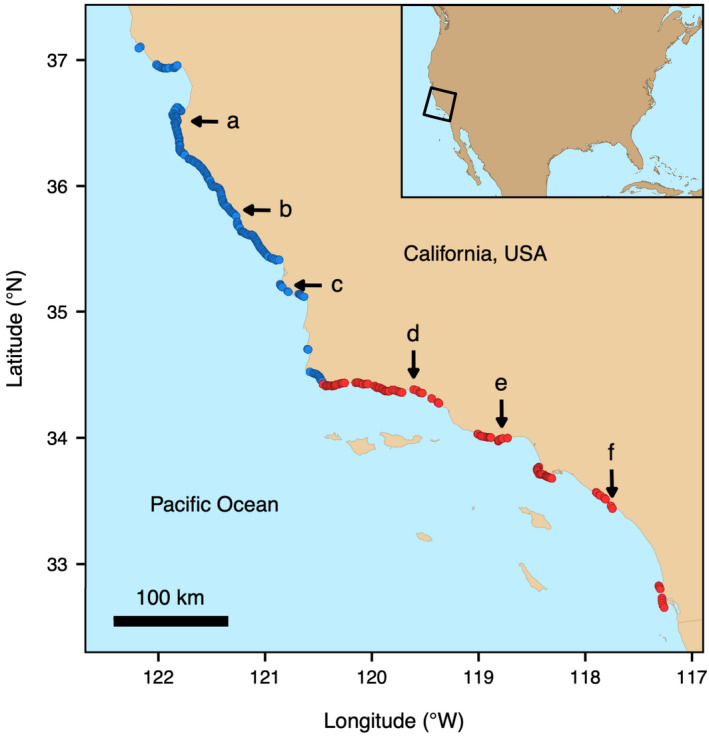
Map of the study region in California, USA, showing the distribution of all persistent mainland giant kelp, where each point location represents a 500‐m segment of coastline (*n* = 361; not to scale). Point Conception divides central California locations (blue) from southern California locations (red). Letters correspond with representative locations plotted in Figure 2

We focused on giant kelp populations along the mainland coast of California from Año Nuevo (37.1°N, 122.3°W) to San Diego (32.5°N, 117.1°W) (Figure 1). Within this range, central California (north of Point Conception; Figure 1) experiences frequent, severe wave disturbances due to exposure to large winter swells and high nutrient availability from upwelling (Bell, Cavanaugh, Reed, & Siegel, 2015; Cavanaugh et al., 2011; Huyer, 1983; Reed et al., 2011). In southern California (south of Point Conception; Figure 1), wave disturbance is more variable, and upwelling is less intense.

### Giant kelp biomass data

**FIGURE 2:**
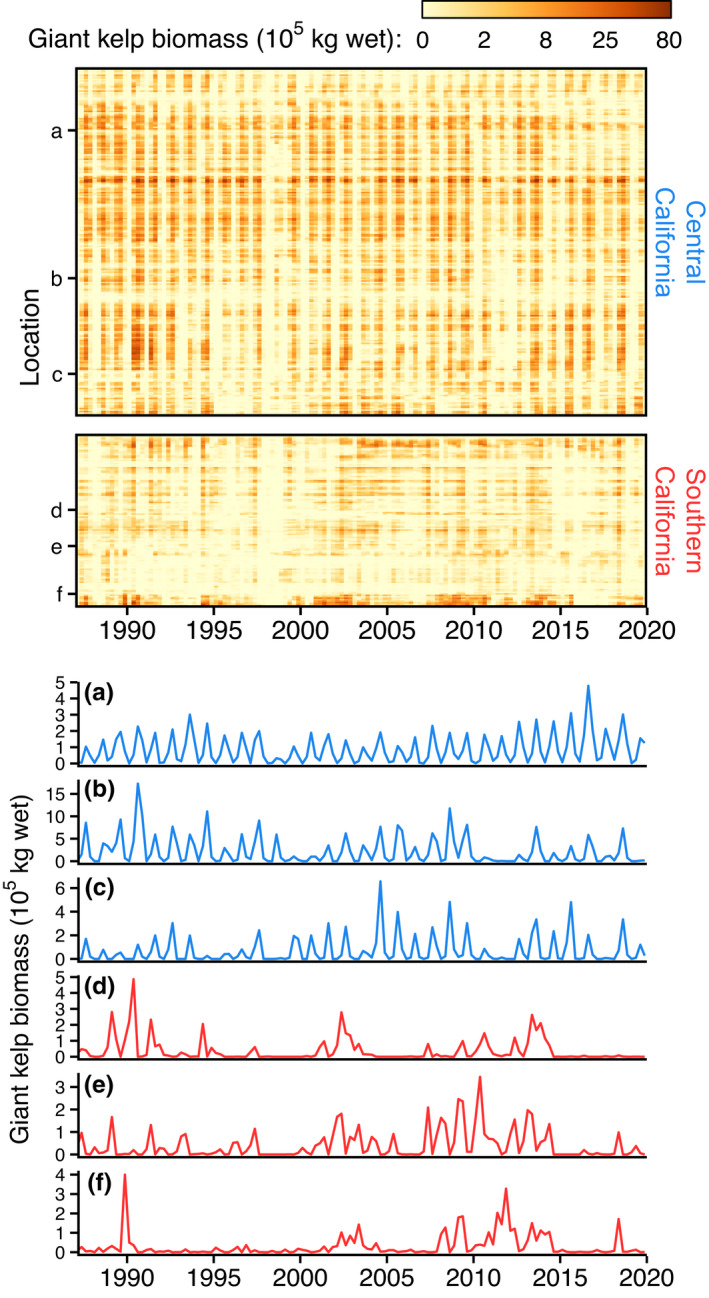
Giant kelp canopy biomass fluctuates synchronously over time, but patterns of synchrony differ by geography and timescale (i.e. period of fluctuations). Top panel shows quarterly giant kelp canopy biomass (log scale) from 1987 through 2019 for all locations (rows) ordered based on alongshore position (see Figure 1). Bottom panel shows biomass time series for three representative locations in each region (indicated by letters in top panel and in Figure 1)

The floating surface canopy formed by giant kelp can be measured by satellite‐based remote sensing (Cavanaugh et al., 2011). We estimated surface canopy biomass (kg wet) from January 1987 through December 2019 using 30‐m resolution multispectral Landsat imagery (details and validation in Cavanaugh et al., 2011; Bell et al., 2020; Bell, Cavanaugh, & Siegel, 2021). These estimates are commonly used as a proxy for population size at local to regional scales (Castorani et al., 2015, 2017; Cavanaugh et al., 2019; Krumhansl et al., 2016; Young et al., 2016). We calculated quarterly mean biomass (winter = January–March; spring = April–June; summer = July–September; autumn = October–December) within 500‐m segments of coastline (hereafter, ‘locations’; number of Landsat measurements per quarter = 2.4 ± 1.2 [mean ± standard deviation]; range = 1–9). We focused on kelp forests that were largely persistent by selecting locations where kelp was not observed (zero biomass or missing data) for no more than 3 years, thereby retaining 83% of the 437 locations for which kelp was present in at least 1 year. This resulted in a 33‐year time series (winter 1987 through autumn 2019) of quarterly mean canopy biomass for 361 locations (Figure 2).

### Oceanographic data

At each location, we estimated quarterly wave and nutrient dynamics from 1987 through 2019. To characterise wave disturbance, we used a time series of coastal swell predictions (detailed methods in Bell, Cavanaugh, Reed, & Siegel, 2015). Briefly, this dataset was generated from a cross‐validated model that combines hourly in situ measurements of significant wave height (hereafter, ‘wave height’) and direction (from the U.S. National Buoy Data Center, NBDC) with real‐time and hindcast swell models (details and validation in Wingeart et al., 2001; Hanson et al., 2009; O'Reilly et al., 2016). We calculated quarterly maximum wave height values for each location across the time series.

In coastal California, daily surface nitrate concentrations can be estimated from sea surface temperature (SST) using established empirical relationships (Palacios et al., 2013; Snyder et al., 2020; Zimmerman & Kremer, 1984). Hence, to quantify nutrient availability, we estimated surface nitrate concentrations from daily SST estimates from 1/4° Advanced Very High‐Resolution Radiometer satellite imagery (details and validation in Reynolds et al., 2007; Bell, Cavanaugh, Reed, & Siegel, 2015; Banzon et al., 2016). We calculated quarterly mean nitrate concentrations (hereafter, ‘nitrate’) for each location across the time series following Snyder et al. (2020).

To evaluate the robustness of estimating kelp nutrient availability from SST, we analysed 38 years (1981–2018) of paired in situ measurements of surface water temperature and nitrate at 0–20 m (the depth range of most giant kelp forests in California; North, 1976) from 83 locations in coastal California sampled by the California Cooperative Oceanic Fisheries Investigations (CalCOFI). Consistent with earlier studies, we found a strong nonlinear negative correlation between these measures (*p* < 0.001, *R* ^2^ = 73%; Appendix S1: Figure S1), justifying the use of SST‐derived nitrate to describe nutrient availability to kelp.

Interannual increases in nitrate availability are positively correlated with the North Pacific Gyre Oscillation (NPGO), a monthly oceanographic climate index that corresponds with large‐scale strengthening of wind‐driven upwelling (Di Lorenzo et al., 2008, 2013; Pennington & Chavez, 2018). Because California giant kelp is positively associated with the NPGO (Bell, Cavanaugh, Reed, & Siegel, 2015), we explored the relationship between kelp synchrony and quarterly mean NPGO values.

### Statistical analyses

We used a wavelet‐based approach to quantify and model the timescale‐specific patterns of kelp synchrony in central and southern California, applying methods first developed in Sheppard et al. (2019). This approach is also implemented and documented in the *wsyn* package (Reuman et al., 2021) in R (R Core Team, 2021). Waves and nitrate were used as predictors in our wavelet model because their influences on giant kelp populations have been well documented (reviewed in Schiel & Foster, 2015). We also performed linear modelling that robustly supported the inclusion of both variables (see Appendix S2). All analyses focused on three ‘timescale bands,’ as introduced in Sheppard et al. (2016, 2019): *annual synchrony* (<2 y period), *short interannual synchrony* (2–4 y period) and *long interannual synchrony* (4–10 y period).

To quantify the spatial synchrony in a region at each time and timescale, we evaluated a wavelet mean field and its time average (mean squared synchrony) at each timescale. Values near 1 indicate high synchrony and values near 0 indicate low synchrony. To test the statistical significance of the timescale‐specific phase agreement among locations in kelp oscillations, we compared the magnitude of their wavelet phasor mean field value to a distribution of possible values that could occur under the null hypothesis of unsynchronised phases. We then applied multivariate wavelet regression models to estimate individual and interactive effects of wave height and nitrate on kelp synchrony, and how these effects varied across geography and timescale. The fraction of the observed mean squared synchrony explained by a model is denoted by *q*_all_ and the contributions of the predictors and their interactions in the multivariate model are denoted by *q*_waves_, *q*_nutrients_ and *q*_int_, for waves, nutrients, and their interaction, respectively. Additional details on the wavelet methods are provided in Appendix S3.

Wavelet model coefficients have both a magnitude and phase, characterising the strength and temporal lag of association respectively. This approach can account for delayed and phase‐shifted associations between the driver and response variables. For each region and timescale band, we report the average phase difference, ϕ, between the fluctuations in each explanatory variable and the corresponding fluctuations in the model of kelp that are attributed to it (this phase shift is given by the phase of the model coefficient). Additional details on phase shifts are provided in Appendix S3: Table S4.

To quantify the spatial synchrony explained by wavelet models, we applied a synchrony attribution theorem (Sheppard et al., 2019). The contributions to synchrony of the predictors are strictly positive (equaling zero when the predictors are themselves spatially unsynchronised), but can exceed 100% of the observed synchrony when offset by negative interaction terms. Positive interaction terms indicate synergistic effects (more synchrony than attributable to the additive effects of independent drivers) and negative terms indicate antagonistic effects (less synchrony than attributable to the additive effects of independent drivers).

Separately, we quantified statistical associations between kelp fluctuations and NPGO fluctuations (reflecting large‐scale interannual nutrient variability) within each region and timescale band. Separating this statistical analysis from analyses using waves and nitrate was necessary because the NPGO index is a non‐spatial representation of large‐scale interannual nutrient variability and thus is correlated with nutrient concentrations at longer timescales (Di Lorenzo et al., 2008, 2013; Pennington & Chavez, 2018). Analyses using spatial predictors (wave height and nitrate) may reflect causal effects of local drivers on local kelp populations, but the NPGO is an index of a large‐scale phenomenon, which can only causally influence kelp dynamics through local covariates. In other words, synchrony statistically ‘explained’ by a widespread statistical association with the NPGO index must result from local mechanisms associated with the NPGO.

## RESULTS

**FIGURE 3:**
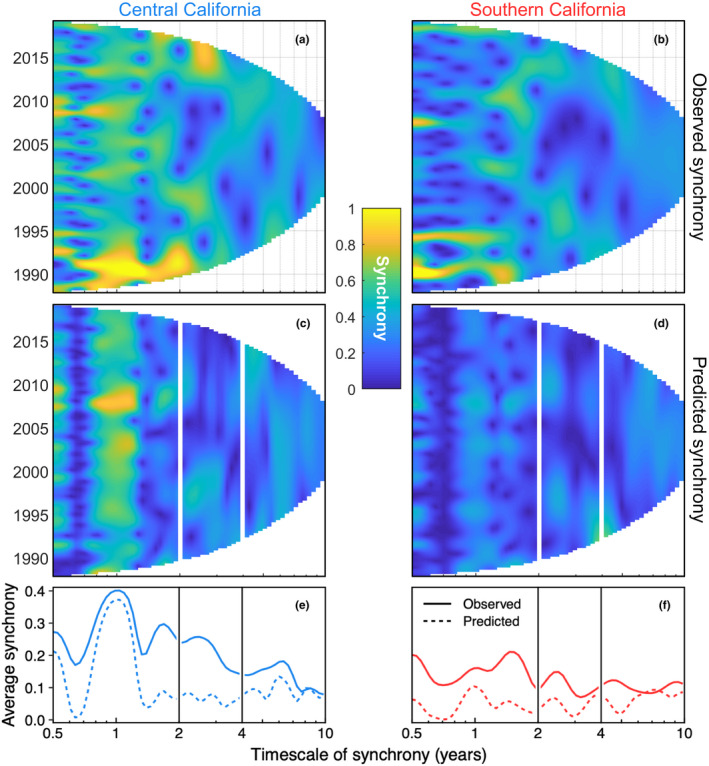
Wavelet mean fields reveal synchrony of giant kelp canopy biomass in central (left) and southern (right) California across time and timescale. Top panels (a–b) show observed synchrony; nearly all features shaded cyan, green, and yellow are highly significant in wavelet phasor mean field testing of phase synchrony (*p* < 0.001; Appendix S3: Figure S3). Middle panels (c–d) show synchrony predicted by wavelet models based on wave disturbance (maximum wave height), nutrient availability (mean nitrate concentration), and their interaction. Bottom panels (e–f) summarise the top and middle panels by averaging observed (solid line) and model‐predicted (dashed line) timescale‐specific synchrony across all years (i.e. the mean squared synchrony), and comparing these observations and model predictions across timescales. Vertical lines separate timescale bands for annual (<2 y period), short interannual (2–4 y period) and long interannual (4–10 y period) synchrony. Note that the *x*‐axis shows the timescale of synchrony on a log scale

**FIGURE 4:**
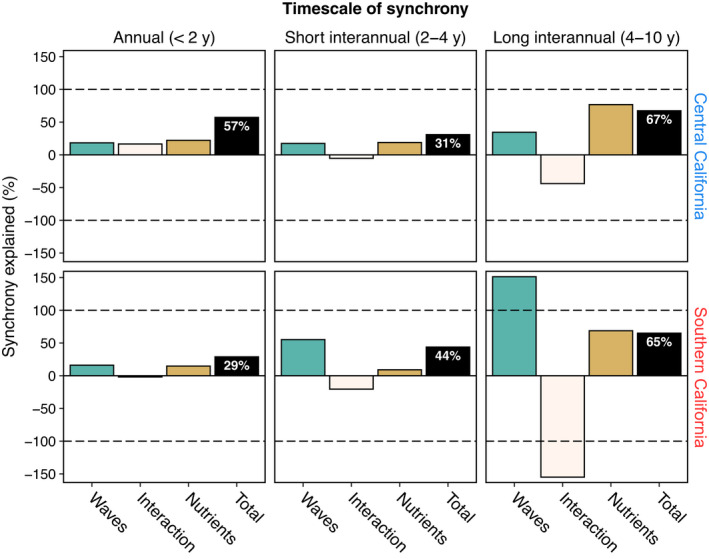
Wave disturbance, nutrient availability, and their interaction explain synchrony in giant kelp canopy biomass across timescales and regions. The black bar shows the total average percentage of synchrony explained by wavelet models (*q*_all_) and coloured bars show the partitioned contributions from maximum wave height (*q*_waves_), mean nitrate concentration (*q*_nutrients_), and their interaction (*q*_int_). For each panel, coloured bars sum to the black bar. Values for the main effects of waves and nutrients are always positive, but can exceed 100% when offset by negative interactions (antagonism). Such antagonistic interactions indicate that the synchronising effects of waves and nutrients counteract one another to reduce synchrony below that attributable to additive effects alone. Likewise, positive interactions indicate synergy between waves and nutrients that enhance synchrony above that attributable to additive effects alone

Addressing *Goal 1*, we quantified the timescale structure of synchrony in giant kelp canopy biomass and its main causes. Wavelet mean fields indicated that the two geographical regions—central and southern California—differ strongly in the dynamics and timescale of kelp synchrony (cf. Figures 3a,b). Annual synchrony (<2 y period) was consistently strong in central California, but generally weak and episodic in southern California (Figures 2, 3). Both regions exhibited moderate and episodic synchrony on short interannual (2–4 y) and long interannual (4–10 y) timescales, but synchrony within both timescale bands was slightly greater in central California (cf. solid lines in Figures 3e,f). Wavelet phasor mean fields demonstrated that for most times and timescales, there was strong among‐location agreement in the phase of kelp oscillations (*p* < 0.001; Appendix S3: Figure S3). Wavelet coherence and wavelet linear modelling revealed that waves, nutrients, and their interaction explained a substantial fraction of kelp synchrony, though the power of these factors to explain synchrony varied by region and timescale (Figure 3c–f; Figure 4 black bars; Table 1 ‘Overall model *q*_all_’; additional details below).

**TABLE 1:** Results of wavelet models predicting giant kelp synchrony from wave disturbance (significant wave height), nutrient availability (surface nitrate concentration), and their interaction. The symbol *q*_all_ denotes the average percentage of synchrony explained by the model across each timescale band; *q*_waves_, *q*_nutrients_, and *q*_int_ denote the partitioned contributions from waves, nutrients, and their interaction, respectively. For main effects, *q* values are strictly positive, but can exceed 100% when offset by negative interaction terms. For interactions, positive and negative *q* values indicate synergism and antagonism, respectively. Phi (ϕ) denotes the average phase difference between each driver and corresponding giant kelp biomass fluctuations, in fractions of π, such that ϕ ≈ 0 indicates an in‐phase relationship, ϕ ≈ ±1 indicates an anti‐phase relationship, and 0 < |ϕ| < 1 indicates lagged relationships (e.g. ϕ = 0.5 indicates a quarter‐cycle phase shift)

	Overall model	Waves		Waves × Nutrients	Nutrients	
	*q*_all_	ϕ	*q*_waves_	*q*_int_	ϕ	*q*_nutrients_
Central California						
Annual (<2 years)	57.1%	−0.84	18.3%	16.6%	0.59	22.1%
Short interannual (2–4 years)	30.9%	−0.65	17.4%	−5.3%	0.01	18.8%
Long interannual (4–10 years)	67.4%	−0.90	34.5%	−43.9%	0.07	76.7%
Southern California						
Annual (<2 years)	28.9%	−0.82	16.1%	−1.9%	0.22	14.7%
Short interannual (2–4 years)	43.8%	−0.73	55.2%	−20.4%	0.03	9.1%
Long interannual (4–10 years)	65.0%	0.59	151.2%	−154.9%	0.83	68.7%

Addressing *Goal 2*, we quantified the importance of interactions between wave and nitrate Moran drivers of kelp synchrony. Contributions of Moran interactions to kelp synchrony were substantial and could be either positive (synergistic) or negative (antagonistic). In central California, waves and nutrients interacted synergistically on annual timescales, producing *q*_int_ = 17% more synchrony than could be attributed to individual effects of these factors; whereas on long interannual timescales (4–10 y period), interactions were strongly antagonistic, diminishing synchrony by *q*_int_ = −44% (Figure 4; Table 1). In southern California, interactions were negative for all timescale bands considered, though much more strongly for long interannual timescales (*q*_int_ = −155%).

Addressing *Goal 3*, our analyses demonstrated that Moran drivers of kelp synchrony and their interactions depended on region and timescale (Figure 4; Table 1). For instance, the aggregate importance of waves, nutrients and their interactions varied across regions and timescales from *q*_all_ = 29% (southern California, <2 y period) to *q*_all_ = 67% (central California, 4–10 y period). Likewise, the main‐effect influence of waves varied from *q*_waves_ = 16% (southern California, <2 y period) to *q*_waves_ = 151% (southern California, 4–10 y period); as did that of nutrients (*q*_nutrients_ = 9%–77%) and interactions (*q*_int_ = −155% to 17%; Table 1). Interactions between Moran drivers had an overwhelming effect on total explained synchrony. For instance, at annual timescales, much more synchrony was explained in central than southern California because interactions were synergistic in central California but negligible in southern California (Table 1).

**FIGURE 5:**
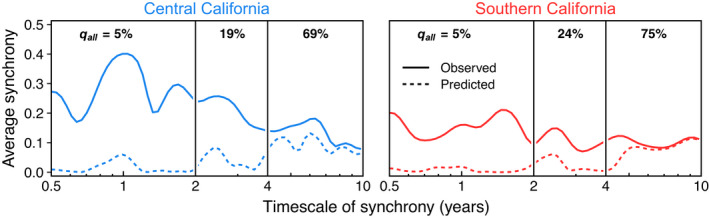
Synchrony of giant kelp canopy biomass is partly explained by fluctuations in the North Pacific Gyre Oscillation (NPGO), an oceanographic climate index that corresponds with large‐scale strengthening of wind‐driven upwelling. Lines and axes as in Figure 3. Numbers at the top of each timescale band show the average percentage of synchrony explained by NPGO wavelet models within a given region and timescale band (*q*_all_; analogous to black bars for total synchrony explained in Figure 4)

Though we emphasise that NPGO can only causally influence giant kelp through local covariates, wavelet models revealed that NPGO was highly coherent with kelp dynamics at long interannual timescales, statistically explaining *q*_all_ = 69%–75% of observed synchrony within the 4–10 y timescale band (Figure 5). NPGO variability was only moderately important to kelp synchrony on short interannual timescales (*q*_all_ = 19%–24%) and not important at annual timescales (*q*_all_ = 5%; Figure 5). The ability of NPGO to statistically explain kelp synchrony was similar between central and southern California regardless of timescale (Figure 5).

## DISCUSSION

Despite the ubiquity and importance of spatial synchrony, resolving how multiple factors interact to determine synchrony across scales in time and space has been a long‐standing challenge (Liebhold et al., 2004; Moran, 1953). Our study of long‐term giant kelp canopy biomass dynamics across California helps narrow this knowledge gap by supporting three major conclusions about environmentally induced synchrony (the Moran effect): (1) Synchrony differs greatly across timescales and geography commensurate with differences in multiple environmental drivers affecting both population decline and growth, such as disturbance and resources. (2) Substantial interactions occur between Moran drivers, which can be synergistic (producing additional synchrony) or antagonistic (reducing synchrony from what would otherwise be expected). (3) The influence of Moran drivers and their interactions differ strongly across timescales and geographical regions, reinforcing the importance of studying synchrony using timescale‐ and geography‐specific approaches (e.g. Defriez et al., 2016; Sheppard et al., 2016; Walter et al., 2017, 2022). These findings represent an important advance because, to our knowledge, interactions among Moran drivers of synchrony have been identified only once previously (Sheppard et al., 2019). Our results also comprise the first example in which disturbance and resources interact to structure synchrony, and the first empirical evidence that Moran effects can interact antagonistically to produce less synchrony than would be expected through additive effects.

How can we intuitively understand the mechanism of interactions between Moran drivers? And how can interactions be synergistic on some timescales and simultaneously antagonistic on others, as we found for giant kelp forests in central California? First, note that large waves have immediate negative effects on kelp biomass, whereas elevated nutrients have positive effects that are delayed by one quarter (Appendix S2) because it takes several weeks to months for new kelp recruitment and growth to reach the water surface and achieve densities detectable from satellites (Bell & Siegel, 2022; Schiel & Foster, 2015). In central California, waves tend to achieve their annual maximum in the winter, whereas nutrients achieve their annual maximum in spring (Bell, Cavanaugh, Reed, & Siegel, 2015). Thus, annual wave effects are negative and occur in winter, whereas annual nutrient effects are positive and manifest (due to growth delays) in summer. So, nutrient and wave effects can temporally align and reinforce each other in producing large annual oscillations: large increases in kelp canopy biomass in summer due to replete nutrients can be followed by major crashes in winter due to large waves. In this scenario, positive interactions between wave and nutrient Moran effects on annual timescales (17%; Table 1) can occur, whenever years with large waves coincide with years with replete nutrients in other locations, a likely common occurrence because both phenomena are related to oceanographic climate. If a large‐wave year in one location coincides with a high‐nutrient year in another location, both locations will tend to have bigger annual kelp oscillations in that year, accentuating annual synchrony. However, sub‐annual timing delays and seasonal differences become negligible when considering long interannual timescales (4–10 y). On such timescales, large‐wave years and nutrient‐replete years counteract each other, so that whenever large‐wave years coincide with nutrient‐replete years in other locations, antagonistic interactions between Moran effects are observed (−44% in central California; Table 1). If a multiannual period of larger‐than‐average waves in one location coincides with a multiannual period of higher‐than‐average nutrients in another location, the interaction between waves and nutrients will tend to reduce kelp canopy biomass in the first location but augment it in the second location, reducing interannual synchrony. Synergistic interactions on annual timescales are not observed in southern California probably because, in that region, variations in waves and nutrients are not as strongly seasonal as in central California and may involve different time lags (Bell, Cavanaugh, Reed, & Siegel, 2015). Using a simple model, Sheppard et al. (2019) further illuminated the mechanisms of interacting Moran effects, showing that the interaction between the effects of two drivers varies in relation to the phase relationship between their effects. However, more analytical modelling is needed to advance a general theory of interacting Moran effects and their effects on synchrony.

We hypothesise that interactions between Moran effects are common and thus argue they should be considered when studying climate effects on synchrony. Both our investigation of kelp forest synchrony and a recent study of synchrony in marine phytoplankton (Sheppard et al., 2019) revealed important interactions between Moran drivers. In our study, interactions between disturbance and resources (nutrients) amplified or dampened kelp synchrony at certain timescales; in Sheppard et al. (2019), synergistic interactions between temperature and predators (grazing zooplankton) enhanced phytoplankton synchrony. We suspect that interacting Moran effects are widespread because multiple, interrelated environmental drivers influence most ecosystems. Hence, future research should quantify between‐variable synchrony for environmental drivers, systematically assess the commonness of interactions between Moran effects, and resolve the potential for climate change to alter such interactions to affect population synchrony and stability (Hansen et al., 2020).

Differences in the geographical setting and time period of prior investigations have contributed to uncertainty about the relative importance of disturbance and resources in structuring kelp forest ecosystems (e.g. Broitman & Kinlan, 2006; Dayton et al., 1992, 1999; Parnell et al., 2010; Reed et al., 2008, 2011, 2016). Our large‐scale, long‐term study helps clarify this debate by supporting the idea that waves and nutrients work together to synchronise giant kelp canopy biomass via influences on kelp loss, recovery, and growth, but that the strength of these synchronising forces varies over space and timescale. Large waves cause massive giant kelp mortality and canopy loss (Bell, Cavanaugh, Reed, & Siegel, 2015; Graham et al., 1997; Reed et al., 2011; Young et al., 2016), and sustained low nutrients reduce kelp recruitment and growth (Deysher & Dean, 1986; Hernández‐Carmona et al., 2001; Kopczak et al., 1991; Zimmerman & Kremer, 1984, 1986); both processes induce kelp synchrony via the Moran effect because they are spatially autocorrelated (Bell, Cavanaugh, Reed, & Siegel, 2015).

Our results also build substantially upon earlier results showing that increasing geographical separation leads to exponential decreases in synchrony (Cavanaugh et al., 2013) by revealing that giant kelp is more synchronous in central than southern California across all timescales, but particularly at annual timescales. These conclusions reinforce prior work demonstrating broad, consistent seasonality of giant kelp canopy biomass in central California—where seasonal wave disturbance (Bell, Cavanaugh, Reed, & Siegel, 2015; Reed et al., 2011) and upwelling of nutrient‐rich water (Huyer, 1983) are more intense—and a lack of consistent giant kelp seasonality in southern California (Bell, Cavanaugh, & Siegel, 2015). Endogenous circannual rhythms related to predictable annual changes in day length (photoperiod) may explain some observed annual synchrony in kelp canopy biomass, but strong geographical differences in the seasonality of kelp biomass (Bell, Cavanaugh, & Siegel, 2015) and the consistency of annual fluctuations (this study) suggest that photoperiod per se probably plays a relatively limited role. Like many plants and macroalgae (Jackson, 2009; Lüning, 2005), giant kelp exhibits annual synchrony in the initiation of reproduction (winter spore production and release; Reed et al., 1997). However, pronounced variation in environmental conditions over space and time can reduce the spatial synchrony and annual consistency of recruitment to adult sporophytes (e.g. Dayton et al., 1992; Deysher & Dean, 1986; Reed et al., 2008; Reed & Foster, 1984). Further study may clarify the role of photoperiod in structuring synchrony across multiple processes (e.g. reproduction, recruitment, growth) that contribute to annual fluctuations in kelp canopy biomass, and how these differ geographically (Bell & Siegel, 2022). Additional research may also reveal how the global generalisability of our results is mediated by giant kelp phenotypic plasticity to diverse oceanographic conditions (Demes et al., 2009). For instance, disturbance may play a more limited role in inducing synchrony among wave‐sheltered populations in southern Chile that exhibit an annual life cycle (Graham et al., 2007).

We found that the North Pacific Gyre Oscillation (NPGO) is a dominant driver of kelp synchrony at interannual timescales, particularly those fluctuations >4 years. NPGO variation corresponds with large‐scale periodic strengthening of coastal nutrient delivery (Di Lorenzo et al., 2008, 2013; Pennington & Chavez, 2018). Thus, it is likely that the power of NPGO in predicting giant kelp synchrony at long interannual timescales (and more modestly at short interannual timescales) manifests mechanistically through nitrate variability. These findings reinforce the important relationship between the NPGO and giant kelp canopy biomass (Bell, Cavanaugh, Reed, & Siegel, 2015), and help clarify the mechanisms by which NPGO variability structures kelp forest dynamics. However, some caution is warranted in interpretation of the strength of synchrony explained by NPGO and its local manifestations (e.g. nutrients) at long interannual timescales due to the potential for model overfitting with relatively few temporal cycles (i.e. about three decadal cycles in our 33‐year time series). Because the NPGO fluctuates predominantly on roughly decadal timescales (Di Lorenzo et al., 2008, 2013), it is not surprising that it was unrelated to annual kelp synchrony. We did not examine other climate indices, such as the El Niño Southern Oscillation or the Pacific Decadal Oscillation, because they are not highly correlated with overall giant kelp biomass dynamics (Bell et al., 2020; Bell, Cavanaugh, Reed, & Siegel, 2015; Cavanaugh et al., 2011). However, the strongest El Niño events on record (1982–1983; 1997–1998; 2015–2016) were associated with widespread giant kelp declines (Cavanaugh et al., 2019; Edwards, 2019). More research is needed, but it may be that typical ENSO variability is not a major driver of synchronous increases or decreases in giant kelp, but extreme ENSO events are important.

Our models could not explain all observed kelp synchrony on any timescale in either region, but predictive power was relatively modest on annual timescales in southern California and on short interannual timescales in both central and southern California. Nevertheless, the positive contributions to synchrony by waves and nutrients, and their antagonistic relationship on multiyear timescales were detectable in the short interannual timescale band. This supports our conceptual model that kelp synchrony is depressed by the correlated but opposing effects of waves and nutrients. Residual synchrony, not explained by these factors, may be due to variables unmeasured in our study, such as species interactions (herbivory, competition) and propagule dispersal. Herbivory may be an important driver of kelp synchrony because grazing sea urchins can denude entire kelp forests (Dayton et al., 1984; Dean et al., 1984; Filbee‐Dexter & Scheibling, 2014), while synchronous sea urchin mortality can reverse state changes (Ebeling et al., 1985; Pearse & Hines, 1979). Sea urchin recruitment in California is highly synchronous on sub‐regional scales (15–100 km; Okamoto et al., 2020), although it remains unclear whether synchronous urchin recruitment leads to synchronous grazing pressure. On the other hand, urchin grazing intensity can vary greatly over short distances (Harrold & Reed, 1985; Rennick et al., 2022) and this may reduce the strength of Moran effects (Bell, Cavanaugh, Reed, & Siegel, 2015). Synchrony may also be diminished by spatial variation in competition between early life stages of giant kelp and benthic algae that suppress kelp recruitment (Beckley & Edwards, 2021; Reed, 1990). Further study of potential synchronising species interactions in kelp forests and possible dependency on abiotic factors (e.g. nutrients, waves) may help explain geographical variation in the synchrony of giant kelp recovery following catastrophic events (Cavanaugh et al., 2019; Edwards, 2004).

Dispersal is another widely accepted mechanism of synchrony (Abbott, 2011; Duncan et al., 2015; Gouhier et al., 2010; Luo et al., 2021) and we argue above that interacting Moran effects may be widespread. However, interactions between dispersal and Moran drivers remain unknown beyond simple theoretical models (Kendall et al., 2000). Giant kelp populations are demographically linked by the passive dispersal of spores by ocean currents, and this process is important to patch colonisation and extinction (Castorani et al., 2015, 2017; Reed et al., 2006; Young et al., 2016). At scales of hundreds of metres, synchronous recruitment after mortality events can produce cohorts with similar age structure (Dayton et al., 1992). Because adult giant kelp sporophytes typically live for 2–3 years (Reed et al., 2008; Rosenthal et al., 1974), the growth and senescence of these cohorts may cause short‐interannual synchrony of kelp canopy biomass in areas not exposed to annual wave disturbance (Bell & Siegel, 2022; Rodriguez et al., 2013). On the other hand, the synchronising effect of dispersal may be interrupted by local processes that inhibit recruitment, such as grazing (Dean et al., 1984; Leonard, 1994), competition (Beckley & Edwards, 2021; Reed & Foster, 1984), or nutrient limitation (Deysher & Dean, 1986; Hernández‐Carmona et al., 2001). The extent to which dispersal synchronises giant kelp over large spatial scales, and whether this effect interacts with local or regional drivers, have not been established.

Our results suggest additional promising directions for future research. Because giant kelp is a foundation species that exerts a strong influence over productivity and biodiversity in kelp forests (Castorani et al., 2018, 2021; Lamy et al., 2020; Miller et al., 2018) and sandy beaches (Dugan et al., 2003; Schooler et al., 2017, 2019), we hypothesise that synchrony of giant kelp and other foundation species may cascade to the community through species interactions (Lee et al., 2020; Morton et al., 2016). This possibility may provide a promising avenue for future research because the cascading effects of population synchrony on community and ecosystem dynamics have been explored in very few systems (Cattadori et al., 2005; Haynes et al., 2009; Kent et al., 2007; Satake et al., 2004; Turkia et al., 2020). Moreover, because synchrony is related to population instability, improved understanding of the patterns and drivers of synchrony in foundation species may help predict how environmental changes influence spatial and temporal ecosystem stability and its ecological services (Kremen, 2005).

## AUTHORS CONTRIBUTION

MCNC, LWS, and DCR conceived the study. MCNC wrote the manuscript. TWB and KCC collected and compiled the data. LWS developed the statistical methods and analysed the data, with substantial contributions from DCR and JAW. All authors contributed to developing the ideas, interpreting the results, and revising the manuscript. All authors gave final approval for publication and declare they have no competing interests.

## Supporting information


Appendix S1
Appendix S2
Appendix S3

## Data Availability

All data and metadata used in this study are publicly available from Bell, Cavanaugh, Reuman, et al. (2021) through the Environmental Data Initiative (https://doi.org/10.6073/pasta/27e795dee803493140d6a7cdc3d23379).
