# Supplementary material for: Disturbance and nutrients synchronise kelp forests across scales through interacting Moran effects
Source: Ecol Lett. 2022 Jun 30;25(8):1854–68. doi: 10.1111/ele.14066 (PMC9541195; doi:10.1111/ele.14066)
Supplement: Supplementary file 1 — Appendix S1 Appendix S2 Appendix S3 [file ELE-25-1854-s001.pdf]

Supporting Information

Disturbance and nutrients synchronise kelp forests across scales through interacting Moran effects.

Max C. N. Castorani[1,*], Tom W. Bell[2,3], Jonathan A. Walter[1], Daniel C. Reuman[4,5,6], Kyle C. Cavanaugh[7], and Lawrence W. Sheppard[4,8]

[1]Department of Environmental Sciences, University of Virginia, Charlottesville, VA 22904 USA

[2]Department of Applied Ocean Physics & Engineering, Woods Hole Oceanographic Institution, Woods Hole, MA 02543 USA

[3]Earth Research Institute, University of California, Santa Barbara, CA 93106 USA

[4]Department of Ecology and Evolutionary Biology, University of Kansas, Lawrence, KS 66047 USA

[5]Kansas Biological Survey and Center for Ecological Research, University of Kansas, Lawrence, KS 66047 USA

[6]Laboratory of Populations, Rockefeller University, New York, NY 10065 USA

[7]Department of Geography, University of California, Los Angeles, CA 90095 USA

[8]Marine Biological Association of the United Kingdom, Plymouth PL1 2PB, UK

***Corresponding author:** Max C. N. Castorani. Department of Environmental Sciences, University of Virginia, Clark Hall, 291 McCormick Rd., P.O. Box 400123, Charlottesville, VA 22904-4123, USA. Tel: 434-243-4949. Fax: 434-982-2137. Email: castorani@virginia.edu

Appendix S1: Figure S1. *In-situ* relationship between sea surface temperature and nitrate. Relationship between paired *in situ* measurements of sea surface water temperature and nitrate concentrations at 0–20 m depth from 83 locations in coastal California sampled by the California Cooperative Oceanic Fisheries Investigations (CalCOFI) from 1981 through 2018. Blue line shows the predicted relationship from a cubic spline generalized additive model (R^2 = 73%, P < 0.001). Data are available from CalCOFI at https://calcofi.org/ccdata/database.html.

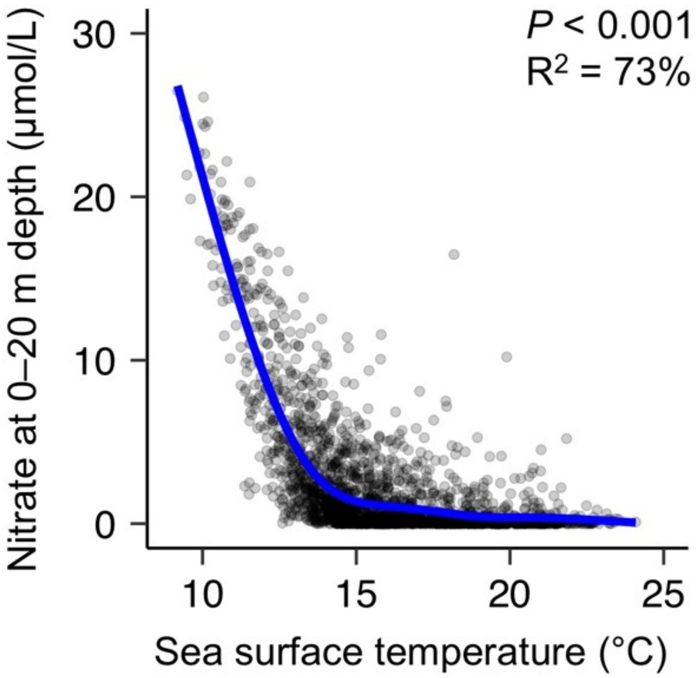

Appendix S2. Supplemental statistical methods.

Although prior research robustly supports that the notion that wave disturbance and seawater nitrate concentrations generally influence giant kelp population dynamics (reviewed in Schiel & Foster 2015), we statistically verified the individual and joint influences of these oceanographic variables on giant kelp canopy biomass in our specific dataset to further justify their subsequent inclusion as predictors in wavelet models (see Methods). We began with correlation analysis of quarterly maximum significant wave height (hereafter, 'wave height') and quarterly mean nitrate concentration (hereafter, 'nitrate'), individually, with giant kelp canopy biomass (hereafter, 'giant kelp'). Using seasonal medians, we gap-filled the 33-year quarterly time series (winter 1987 through autumn 2019) of giant kelp, wave height, and nitrate. We then demeaned and detrended all time series (see Methods). Next, for each location, we computed the temporal correlation between time series of giant kelp and time series of nitrate lagged by 0, 1, 2, 3, or 4 quarters. We repeated this procedure for wave height. Then, for each oceanographic variable and time lag, we averaged the resulting correlation coefficients across all locations to produce spatially averaged lagged correspondences between giant kelp and wave height, and giant kelp and nitrate.

We significance tested the spatially averaged lagged correlations using the following randomization procedure. We generated 1000 randomized 'surrogate' datasets of wave height and nitrate, each consisting of a 33-year quarterly time series at 361 locations; this approach preserved the spatial and temporal autocorrelation properties of the original datasets but yielded surrogate data statistically unrelated to the giant kelp time series (see details below). Next, using the methods described above, we calculated the spatially averaged correlation between the giant kelp data and each surrogate dataset of wave height or nitrate. For each oceanographic variable,

we then computed the quantile, ρ_i, of each empirically based statistic from the corresponding distribution of surrogate-based statistics. This quantile provides information about the statistical significance of correlation at significance level α: $\rho_i > 1 - \alpha/2$ indicates significant positive correlation between giant kelp and the oceanographic variable, and $\rho_i < \alpha/2$ indicates significant negative correlation.

Surrogate data represent the null hypothesis that time series of wave height or nitrate are unrelated to giant kelp. It was essential that surrogate datasets preserve spatiotemporal autocorrelations because null distributions of our correlation statistics in the absence of relationships between giant kelp and oceanographic variables can depend markedly on autocorrelation features of the data. Surrogates were generated using the "amplitude adjusted Fourier surrogates" (AAFT) method described by Prichard & Theiler (1994) and Schreiber & Schmitz (2000), implemented in the R package *wsyn* (Reuman *et al.* 2021). The AAFT method is one of several related techniques which are standard in some fields of application and have recently been applied several times in ecology (Sheppard *et al.* 2016, 2017, 2019, 2020; Walter *et al.* 2017, 2020, 2021; Anderson *et al.* 2019). Surrogate datasets for wave height and nitrate were based on demeaned, but not detrended, time series. Surrogate time series themselves were then detrended prior to analysis, providing congruity with the computation of empirical correlation statistics and improving statistical correspondence with real time series. Similar analyses were carried out using annualized time series of giant kelp, wave height, and nitrate (i.e., for each oceanographic variable, we averaged the four quarters in each calendar year).

Results from analysis of quarterly data (Table S1) show strong evidence for significant positive correlation between giant kelp and nitrate, with giant kelp lagging nitrate by one quarter. Giant kelp was also significantly negatively related to wave height with no lag. These results

match our *a priori* predictions because large waves break or dislodge adult giant kelp (Seymour *et al.* 1989) to cause massive and immediate biomass losses (Dayton *et al.* 1992; Graham *et al.* 1997; Reed *et al.* 2011). Likewise, high nitrate concentrations promote giant kelp recruitment (Deysher & Dean 1986; Hernández-Carmona *et al.* 2001) and growth (Zimmerman & Kremer 1984, 1986; Kopczak *et al.* 1991), but there may be a delay of several weeks to months before substantial biomass reaches the water surface and achieves densities detectable by 30 m resolution Landsat satellite remote sensing (Bell *et al.* 2015). Results from analyses of annualized data (Table S2) show significant ($P < 0.05$) or marginally significant ($P < 0.1$) zero-lag positive correlations between annual mean nitrate and annual mean giant kelp, and zero-lag negative correlations between annual mean wave height and annual mean giant kelp.

The correlation results presented are consistent with earlier work on the effects of wave disturbance and nutrient availability on giant kelp canopy biomass as measured using Landsat (Cavanaugh *et al.* 2011; Bell *et al.* 2015; Young *et al.* 2016). However, we were also interested in whether the statistical influence of each of these oceanographic variables on giant kelp would still be detectable when controlling for the influence of the other variable. In other words, because both wave height and nitrate are related to common oceanographic processes, and, historically, multiannual periods of low nutrients have often coincided with large wave events (Dayton *et al.* 1992, 1999; Parnell *et al.* 2010; Schiel & Foster 2015), it is not clear, *a priori*, that the two influences described above are distinct. Hence, to justify our subsequent inclusion of both wave height and nitrate in our wavelet models, we investigated whether both variables are statistically necessary to explain giant kelp dynamics. We first generated 1000 joint nitrate-wave height surrogate datasets; each surrogate dataset consists of quarterly time series of wave height and nitrate for each of 361 sampling locations, generated using the AAFT algorithm to preserve

spatial and temporal autocorrelation within each variable, and spatial and temporal cross correlations between the variables, but also to destroy through randomization any potential relationship between the oceanographic variables and giant kelp. Surrogates were again generated based on demeaned, but not detrended, empirical time series, and then the surrogate timeseries themselves were detrended. Next, for each sampling location, we regressed giant kelp at time t against wave height at time t and nitrate at time $t - 1$, obtained the coefficient of determination of the regression, and averaged these values across sampling locations. We also computed this same spatially averaged coefficient of determination statistic for each joint surrogate dataset of the two oceanographic variables. We calculated the quantile of the empirically based statistic in the corresponding distribution of surrogate-based statistics, which provides information about whether wave height and nitrate, jointly, are significant predictors of giant kelp dynamics.

We carried out similar computations using surrogates of wave height only (using empirical time series of nitrate in these regressions) or using surrogates of nitrate only (using empirical time series of wave height in these regressions). In both cases, similar quantiles were obtained, providing information about whether wave height or nitrate give additional, statistically significant explanatory power of giant kelp dynamics beyond the explanatory power of the other variable. Analogous regression analyses were carried out using annualized data, with lags of zero used for time series of both wave height and nitrate.

Results from analyses of quarterly and annualized data (Table S3) showed strong evidence for the independent effects of wave height and nitrate on giant kelp biomass dynamics. In other words, there is strong justification to retain both variables in statistical models.

Appendix S3. Supplemental wavelet methods.

The approach used here follows closely the approach used to decompose wavelet synchrony of plankton time-series in Sheppard *et al.* (2019), where certain definitions and formulas were introduced for the first time.

Prior to analyses, time series of all variables were gap-filled with seasonal medians, demeaned (centered), and linearly detrended. Time series were subjected to a complex continuous Morlet wavelet transform (details in Sheppard *et al.* 2016). We quantified the strength of synchrony among the locations within each region using the magnitude of the *wavelet mean field* (WMF) at each time and timescale. The time-average squared magnitude of the WMF, called the mean squared synchrony, is a quantity between 0 and 1 expressing the synchrony at each timescale, which can be compared to the synchrony expected given an observed relationship with a spatially synchronous predictor. We also evaluated the *wavelet phasor mean field* (WPMF) at each time and timescale, comparing empirical values against the distribution expected for n random phases (n = no. of locations, 1000 realizations; details in Anderson *et al.* 2019), to establish that spatial synchrony was significant across time and timescales. Mean squared synchrony is equivalent to the wavelet power of the average kelp time series (across all locations) divided by the average of all wavelet power values for each individual kelp time series. Both the WMF and the WPMF show the strength of synchrony as a function of time and timescale; while the WMF quantifies synchrony including both phase synchrony and correlations in the magnitude of the synchronized fluctuations, the WPMF shows only phase synchrony, but allows for significance testing.

To identify the relative importance of waves, nitrate, and their interaction in inducing kelp synchrony within each region and timescale band, we used a multivariate linear modeling

approach for wavelet transforms, creating model-predicted wavelet transform values for each location, time, and timescale as the sum of contributions by waves and nitrate.

At each timescale we obtained a best-fit coefficient for each predictor by maximizing the wavelet power explained across all locations within each region, analogous to maximizing the coefficient of determination (R^2) in ordinary least-squares regression (Sheppard *et al.* 2019).

In the main text, we report the observed average phase shifts, ϕ, between the fluctuations in the proposed drivers, waves and nutrients, and the fluctuations in kelp attributed to them. These phase shifts are obtained from the model coefficients. We also checked the average phase difference between driver and kelp transforms for comparison, as in Sheppard *et al.* (2019) Appendix S10, which we called ϕ' (see Table S4). We averaged over each timescale band as standard for circular statistics. The phase relationship, ϕ, between kelp and nutrients in central California was found to be an approximately quarter cycle lag at the annual timescale, with near zero lag at longer timescales. This implies that, for this region, kelp canopy biomass peaks shortly after nutrient fluctuations. In southern California, the lag at annual timescales is somewhat less (0.11 cycles on average), there is minimal lag at short interannual timescales (2–4 y), but a near half cycle lag (i.e., antiphase relationship) at long interannual timescales (4–10 y). The relationship between waves and kelp is generally near antiphase: phase differences in all bands in both central California and southern California were always greater than a quarter cycle, indicating that peaks in waves are associated with troughs in kelp fluctuations.

When comparing the phase shift obtained from the two-driver model (ϕ) to the average phase differences between the transforms themselves (ϕ') at annual and short interannual timescales (2–4 y) the agreement was generally good, however at long interannual timescales (4–10 y) the values can be very different. Waves and nutrients are themselves coherent on long

interannual timescales, as demonstrated by their large interaction term in the attribution of synchrony (see below). This collinearity raises the possibility of inaccurate or overfitted coefficients and inaccurate estimates of the true phase relationships and synchrony explained at long interannual timescales. The anticipated phase shifts are not always observed at the longest timescales.

Having obtained model coefficients, it is possible to represent the mean squared synchrony of the kelp wavelet transform at a given timescale, σ, in terms of contributions due to the synchrony of the model, which we call $q(\sigma)$, given in Sheppard *et al.* (2019) Appendix S15, equation 47. The denominator and numerator of this expression represent observed mean squared synchrony and the model prediction, respectively. Here, we report values of q for timescale bands, q_{all}, obtained by averaging both the numerator (contribution of the model) and denominator (mean squared synchrony) in the expression for $q(\sigma)$ over timescales in the band, prior to taking the quotient. Such an attribution requires that synchrony due to cross terms between model effects and model residuals at different locations can be neglected. At a given timescale, the contribution of cross terms is given by Sheppard *et al.* (2019) Appendix S15, equation 47. We averaged these cross terms over each band in the same way as for $q(\sigma)$ and in no band did they exceed 10%.

The fraction of synchrony explained by the model can itself be partitioned into $q_k(\sigma)$ for $k = 1$, $k = 2$, and $k = q_{int}(\sigma)$. These three quantities represent the contribution to synchronous fluctuations of the first predictor ($k = 1$), the second predictor ($k = 2$), and the interaction between the predictors ($q_{int}(\sigma)$), respectively, and are given in Sheppard *et al.* (2019) Appendix S15, equation 62 and equation 63. We averaged over each band in the same way as for $q(\sigma)$ to report the synchrony attributable to waves (q_{waves}), nitrate ($q_{nitrates}$), and their interaction (q_{int}). These

three contributions to synchrony sum to $q(\sigma)$ for any given timescale σ, and the same is true of the band-average values.

The interaction terms in the spatial synchrony predicted by the wavelet model can be positive (synergistic) or negative (antagonistic) if the contribution to the wavelet model made by one predictor variable reinforces or counteracts that of another. Locally, this increases or reduces the net variability contributed by the predictors. Regionally, it increases or reduces the net spatially synchronous variability contributed by spatially synchronous predictors.

At long timescales the effects of waves and nutrients on kelp synchrony are generally antagonistic. In southern California, at the longest timescales, q associated with waves (q_{waves}) exceeds the observed synchrony, but q_{int} is also large and negative, and therefore the net synchrony explained by the model of waves and nutrients (q_{all}) is only 65%. This represents an extreme example of the same pattern seen elsewhere. The high collinearity of wave and nutrient variability apparent at this timescale may result in some overfitting in the wavelet models, but we may also interpret this result as indicating that, hypothetically, the long term spatial synchrony of kelp might be higher than currently observed if the effects of wave action were as observed but those of nutrient variability were somehow absent.

References

Anderson, T.L., Sheppard, L.W., Walter, J.A., Hendricks, S.P., Levine, T.D., White, D.S., *et al.* (2019). The dependence of synchrony on timescale and geography in freshwater plankton. *Limnol. Oceanogr.*, 64, 483–502.

Bell, T.W., Cavanaugh, K.C., Reed, D.C. & Siegel, D.A. (2015). Geographical variability in the controls of giant kelp biomass dynamics. *J. Biogeogr.*, 42, 2010–2021.

Cavanaugh, K.C., Siegel, D.A., Reed, D.C. & Dennison, P.E. (2011). Environmental controls of giant kelp biomass in the Santa Barbara Channel, California. *Mar. Ecol. Prog. Ser.*, 429, 1–17.

Dayton, P.K., Tegner, M.J., Edwards, P.B. & Riser, K.L. (1999). Temporal and spatial scales of kelp demography: the role of oceanographic climate. *Ecol. Monogr.*, 69, 219–250.

Dayton, P.K., Tegner, M.J., Parnell, P.E. & Edwards, P.B. (1992). Temporal and spatial patterns of disturbance and recovery in a kelp forest community. *Ecol. Monogr.*, 62, 421–445.

Deysher, L.E. & Dean, T.A. (1986). In situ recruitment of sporophytes of the giant kelp, *Macrocystis pyrifera* (L.) C.A. Agardh: effects of physical factors. *J. Exp. Mar. Biol. Ecol.*, 103, 41–63.

Graham, M.H., Harrold, C., Lisin, S., Light, K., Watanabe, J.M. & Foster, M.S. (1997). Population dynamics of giant kelp *Macrocystis pyrifera* along a wave exposure gradient. *Mar. Ecol. Prog. Ser.*, 148, 269–279.

Hernández-Carmona, G., Robledod, D. & Serviere-Zaragozab, E. (2001). Effect of nutrient availability on *Macrocystis pyrifera* recruitment and survival near its southern limit off Baja California. *Bot. Mar.*, 44, 221–229.

Kopczak, C.D., Zimmerman, R.C. & Kremer, J.N. (1991). Variation in nitrogen physiology and growth among geographically isolated populations of the giant kelp, *Macrocystis pyrifera* (Phaeophyta). *J. Phycol.*, 27, 149–158.

Parnell, P.E., Miller, E.F., Lennert-Cody, C.E., Dayton, P.K., Carter, M.L. & Stebbins, T.D. (2010). The response of giant kelp (*Macrocystis pyrifera*) in southern California to low-frequency climate forcing. *Limnol. Oceanogr.*, 55, 2686–2702.

Prichard, D. & Theiler, J. (1994). Generating surrogate data for time series with several simultaneously measured variables. *Phys. Rev. Lett.*, 73, 951–954.

Reed, D.C., Rassweiler, A., Carr, M.H., Cavanaugh, K.C., Malone, D.P. & Siegel, D.A. (2011). Wave disturbance overwhelms top-down and bottom-up control of primary production in California kelp forests. *Ecology*, 92, 2108–2116.

Reuman, D.C., Anderson, T.L., Walter, J.A., Zhao, L. & Sheppard, L.W. (2021). wsyn: Wavelet Approaches to Studies of Synchrony in Ecology and Other Fields. R package version 1.0.4. http://CRAN.R-project.org/package=wsyn.

Schiel, D.R. & Foster, M.S. (2015). *The Biology and Ecology of Giant Kelp Forests*. University of California Press, Berkeley, California, USA.

Schreiber, T. & Schmitz, A. (2000). Surrogate time series. *Phys. Nonlinear Phenom.*, 142, 346–382.

Seymour, R.J., Tegner, M.J., Dayton, P.K. & Parnell, P.E. (1989). Storm wave induced mortality of giant kelp, *Macrocystis pyrifera*, in Southern California. *Estuar. Coast. Shelf Sci.*, 28, 277–292.

Sheppard, L.W., Bell, J.R., Harrington, R. & Reuman, D.C. (2016). Changes in large-scale climate alter spatial synchrony of aphid pests. *Nat. Clim. Change*, 6, 610–613.

Sheppard, L.W., Defriez, E.J., Reid, P.C. & Reuman, D.C. (2019). Synchrony is more than its top-down and climatic parts: interacting Moran effects on phytoplankton in British seas. *PLOS Comput. Biol.*, 15, e1006744–e1006744.

Sheppard, L.W., Mechtley, B., Walter, J.A. & Reuman, D.C. (2020). Self-organizing cicada choruses respond to the local sound and light environment. *Ecol. Evol.*, 10, 4471–4482.

Sheppard, L.W., Reid, P.C. & Reuman, D.C. (2017). Rapid surrogate testing of wavelet coherences. *EPJ Nonlinear Biomed. Phys.*, 5, 1–9.

Walter, J.A., Hallett, L.M., Sheppard, L.W., Anderson, T.L., Zhao, L., Hobbs, R.J., *et al.* (2021). Micro-scale geography of synchrony in a serpentine plant community. *J. Ecol.*, 109, 750–762.

Walter, J.A., Sheppard, L.W., Anderson, T.L., Kastens, J.H., Bjørnstad, O.N., Liebhold, A.M., *et al.* (2017). The geography of spatial synchrony. *Ecol. Lett.*, 20, 801–814.

Walter, J.A., Sheppard, L.W., Venugopal, P.D., Reuman, D.C., Dively, G., Tooker, J.F., *et al.* (2020). Weather and regional crop composition variation drive spatial synchrony of lepidopteran agricultural pests. *Ecol. Entomol.*, 45, 573–582.

Young, M.A., Cavanaugh, K.C., Bell, T.W., Raimondi, P.T., Edwards, C.A., Drake, P.T., *et al.* (2016). Environmental controls on spatial patterns in the long-term persistence of giant kelp in central California. *Ecol. Monogr.*, 86, 45–60.

Zimmerman, R.C. & Kremer, J.N. (1984). Episodic nutrient supply to a kelp forest ecosystem in Southern California. *J. Mar. Res.*, 42, 591–604.

Zimmerman, R.C. & Kremer, J.N. (1986). *In situ* growth and chemical composition of the giant kelp, *Macrocystis pyrifera*: response to temporal changes in ambient nutrient availability. *Mar. Ecol. Prog. Ser.*, 27, 277–285.

Appendix S2: Table S1. Spatially averaged lagged correlations between quarterly wave height or nitrate time series and quarterly giant kelp time series, and the significance of these correlations compared to randomized surrogate datasets. A positive spatial average correlation value and a quantile value greater than 1 – α/2 indicates significant positive correlation, at the given lag, between giant kelp and the oceanographic variable (with significance level α). Likewise, a negative spatial average correlation value and a quantile value less than α/2 indicates significant negative correlation, at the given lag, between giant kelp the oceanographic variable.

Oceanographic variable	Lag (quarters)	Spatial average correlation	Quantile in surrogate values of the same statistic
**Wave height**	**0**	**–0.361**	**0.001**
Wave height	1	–0.038	0.447
Wave height	2	0.250	0.843
Wave height	3	–0.024	0.501
Nitrate	0	0.017	0.506
**Nitrate**	**1**	**0.386**	**1.000**
Nitrate	2	–0.002	0.544
Nitrate	3	–0.238	0.099

Note: Bold face indicates significant correlations at α = 0.05.

Appendix S2: Table S2. Spatially averaged lagged correlations between annual mean wave height or nitrate time series and annual mean giant kelp time series, and the significance of these correlations compared to randomized surrogate datasets. A positive spatial average correlation value and a quantile value greater than 1 – α/2 indicates significant positive correlation, at the given lag, between giant kelp and the oceanographic variable (with significance level α). Likewise, a negative spatial average correlation value and a quantile value less than α/2 indicates significant negative correlation, at the given lag, between giant kelp the oceanographic variable.

**Oceanographic variable**	**Lag (years)**	**Spatial average correlation**	**Quantile in surrogate values of the same statistic**
**Wave height**	**0**	**–0.201**	**0.012**
Wave height	1	0.007	0.539
Wave height	2	0.068	0.758
Wave height	3	0.146	0.947
**Nitrate**	**0**	**0.155**	**0.953**
Nitrate	1	0.106	0.856
Nitrate	2	0.097	0.816
Nitrate	3	0.111	0.854

Note: Bold face indicates significant correlations at α = 0.05.

Appendix S2: Table S3. Lagged regression models of giant kelp dynamics and their significance compared to models using surrogate nitrate and wave height datasets as predictor variables. Quarterly giant kelp data were regressed against quarterly nitrate data with a 1-quarter lag and quarterly wave data with no lag. For annual mean data, no lags were used for either variable. Regressions were carried out on a location-by-location basis. R^2 denotes the spatially averaged model coefficient of determination. Quantiles are shown for the empirically based R^2 relative to the distribution of surrogate-based R^2 values computed using (a) surrogate data for wave height (but original data for nitrate), (b) surrogate data for nitrate (but original data for wave height), or (c) surrogate data for both wave height and nitrate. For both quarterly and annual data, models with the original data for wave height and nitrate performs better than all surrogate data in 100% (1000/1000) of simulations.

		Quantile in surrogate data		
	R^2	(a) Wave height	(b) Nitrate	(c) Wave height and nitrate
Quarterly data	0.256	**0.985**	**1.000**	**1.000**
Annual data	0.169	**0.996**	**0.988**	**1.000**

Note: Bold face indicates quantile values > 0.950, in which surrogated variables explain a significant portion of giant kelp dynamics at $\alpha = 0.05$.

Appendix S3: Figure S3. Wavelet mean fields with significance contours. Wavelet mean fields (WMFs) showing the synchrony of giant kelp canopy biomass within central California (left) and southern California (right) across time and timescale (note log scale), overlaid with significance contours from wavelet phasor mean field (WPMF) testing. Top and bottom panels show observed and model-predicted synchrony, respectively. Areas *outside* of ellipsoids represent significant WPMF phase magnitudes at $P < 0.1$ (dotted line), $P < 0.05$ (dot-dash line), $P < 0.01$ (dashed line), and $P < 0.001$ (solid line) relative to a null hypothesis of no association between phases (i.e., ellipsoids enclose low-significance features).

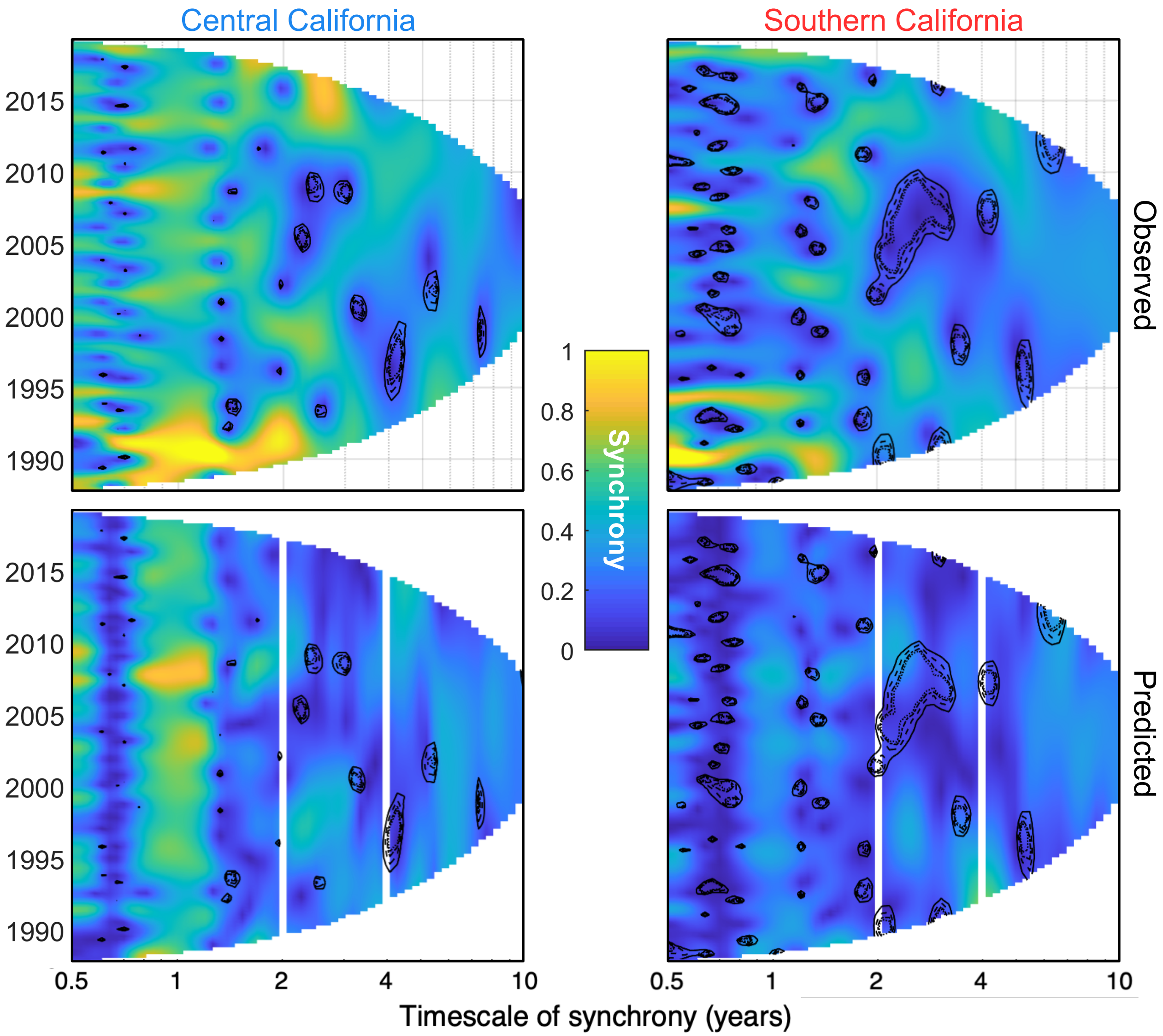

318

Appendix S3: Table S4. Results of wavelet models predicting giant kelp synchrony from wave disturbance (significant wave height), nutrient availability (surface nitrate concentration), and their interaction. Phi (ϕ) denotes the average phase difference between each driver and corresponding giant kelp biomass fluctuations in our bivariate model, in fractions of π, such that ϕ ≈ 0 indicates an in-phase relationship, ϕ ≈ ±1 indicates an anti-phase relationship, and 0 < |ϕ| < 1 indicates lagged relationships (e.g., ϕ = 0.5 indicates a quarter-cycle phase shift). For comparison we present ϕ', the average phase difference between the kelp and driver transforms; this is the phase associated with the driver's coefficient in a one-driver model.

	Waves		Nutrients	
	ϕ	ϕ'	ϕ	ϕ'
**Central California**				
Annual (< 2 y)	–0.84	-0.86	0.59	0.53
Short interannual (2–4 y)	–0.65	-0.89	0.01	0.05
Long interannual (4–10 y)	–0.90	0.43	0.07	0.16
**Southern California**				
Annual (< 2 y)	–0.82	-0.94	0.22	0.18
Short interannual (2–4 y)	–0.73	-0.73	0.03	0.06
Long interannual (4–10 y)	0.59	0.72	0.83	0.55
